# Evaluation of the gastric microbiota based on body mass index using 16S rRNA gene sequencing

**DOI:** 10.3389/fcimb.2025.1651316

**Published:** 2025-09-09

**Authors:** Sang Hoon Lee, Eun Bae Kim, Sung Chul Park, Seung-Joo Nam, Hyunseok Cho, Han Jo Jeon, Sang Pyo Lee

**Affiliations:** 1 Department of Internal Medicine, Kangwon National University College of Medicine, Chuncheon, Republic of Korea; 2 Department of Applied Animal Science, Kangwon National University College of Animal Life Sciences, Chuncheon, Republic of Korea; 3 Institute of Animal Life Science, Kangwon National University, Chuncheon, Republic of Korea; 4 Department of Pediatrics, Kangwon National University College of Medicine, Chuncheon, Republic of Korea; 5 Department of Internal Medicine, Korea University College of Medicine, Seoul, Republic of Korea; 6 Department of Internal Medicine, Hanyang University College of Medicine, Seoul, Republic of Korea

**Keywords:** body mass index, gastric microbiota, obesity, 16S rRNA sequencing, metabolic dysregulation

## Abstract

**Introduction:**

Obesity is a multifactorial condition influenced by various factors, including the gut microbiota. However, the relationship between the gastric microbiota and obesity remains poorly understood. This study aimed to investigate the composition of gastric microbiota, excluding *Helicobacter pylori*, in relation to body mass index (BMI) and metabolic indicators.

**Methods:**

Thirty participants undergoing health checkups were classified into three groups—normal weight (BMI 18.5–22.9), overweight (BMI 23.0–24.9), and obese (BMI ≥25.0)—with ten individuals per group. Those with *H. pylori* infection, atrophic gastritis, or intestinal metaplasia were excluded. Gastric microbiota from four antral biopsies per subject were analyzed using 16S rRNA sequencing and functional profiling by metagenomic prediction.

**Results and discussion:**

Alpha diversity (Gini–Simpson index) was significantly lower in the combined overweight/obese group than that in the normal group (*P*=0.049). Beta diversity analysis revealed clear group separation (Bray–Curtis, *P*=0.005; unweighted UniFrac, *P*=0.004). Significant species differences between the groups were observed; specifically, the abundances of *Muribaculum gordoncarteri*, *Turicibacter bilis*, and *Duncaniella dubosii*, were significantly reduced in the overweight/obese group. Functional predictions showed differential enrichment of pathways related to fatty acid, amino acid, vitamin, and carbohydrate metabolism across BMI categories. These findings suggest that alterations in the gastric microbiota may be linked to obesity and metabolic dysregulation.

## 1 Introduction

Obesity has become a global health issue due to its association with numerous comorbidities, including type 2 diabetes, metabolic dysfunction-associated steatotic liver disease, cardiovascular diseases, and certain cancers (Fall et al., 2017). The prevalence of obesity is increasing, prompting considerable interest in understanding its underlying mechanisms and contributing factors (Afshin et al., 2017).

Obesity is a complex multifactorial disease with diverse etiologies, including genetics, lifestyle, and socioeconomic, and environmental risk factors (Ghosh and Bouchard, 2017). Among these risk factors, the role of gut microbiota in the pathogenesis of obesity has gained substantial attention through various studies (Ley et al., 2005; Turnbaugh et al., 2006; Maruvada et al., 2017). Alterations in gut microbial composition, known as dysbiosis, have been implicated in metabolic disturbances that exacerbate obesity-related conditions​. Specifically, obesity has been associated with a decrease in the relative abundance of beneficial bacterial species, such as Bacteroidota (formerly known as Bacteroidetes) and an increase in Bacillota (formerly known as Firmicutes) (Ley et al., 2005; Turnbaugh et al., 2006). Studies using next-generation sequencing techniques, such as 16S rRNA sequencing have revealed that these microbial changes affect energy harvesting, lipid metabolism, and systemic inflammation, thereby contributing to obesity.

While most research has focused on the intestinal microbiota, other regions of the gastrointestinal tract, such as the stomach, also harbor distinct microbial communities that may influence metabolic health (Engstrand and Graham, 2020). *Helicobacter pylori* has been suggested to be associated not only with various gastrointestinal diseases but also with obesity and metabolic disorders (Azami et al., 2021; Baryshnikova et al., 2024). However, the gastric microbiota, except *H. pylori* infection, remain understudied in relation to obesity. A previous study in mice reported that high-fat diets induced dysbiosis not only in the intestinal microbiota but also in the gastric microbiota, potentially affecting the development and progression of metabolic diseases (He et al., 2018). This study aimed to investigate the gastric microbiota composition according to body mass index (BMI) categories and their potential associations with metabolic indices in humans. By analyzing gastric mucosal samples using 16S rRNA sequencing and functional profiling, this study seeks to bridge the gap in understanding the relationship between gastric microbiota (except *H. pylori*) and obesity beyond the established roles of intestinal microbiota.

## 2 Materials and methods

### 2.1 Study design and population

This study was a cross-sectional observational analysis conducted at three medical centers in South Korea: Kangwon National University Hospital, Korea University Anam Hospital, and Hallym University Dongtan Sacred Heart Hospital. Thirty participants, aged 20 to 65 years, with no upper gastrointestinal symptoms were recruited between December 2021 and November 2023. Participants were categorized into three groups (*n*=10) based on BMI: normal weight (18.5–22.9 kg/m²), overweight (23.0–24.9 kg/m²), and obese (≥25.0 kg/m²). Participants were excluded if they: (1) did not consent to participate in the study; (2) were pregnant or breastfeeding; (3) had serious underlying diseases such as heart disease, renal failure, or liver cirrhosis (excluding hypertension, diabetes, and dyslipidemia); (4) had taken proton pump inhibitors or H2-blockers within the past 4 weeks; (5) had taken antibiotics or probiotics within the past 4 weeks; (6) were underweight with a BMI <18.5; (7) tested positive for *H. pylori* or had previously received *H. pylori* eradication therapy; (8) had atrophic gastritis or intestinal metaplasia detected on upper endoscopy; (9) had undergone previous gastric surgery; (10) had a prior diagnosis of gastric cancer; (11) had a history of acute cerebrovascular or cardiovascular events within the past 3 months; (12) had human immunodeficiency virus or active tuberculosis; or (13) were otherwise deemed unsuitable for participation by the investigators.

Written informed consent was obtained from all the participants. The study was approved by the Institutional Review Board of Kangwon National University Hospital (KNUH B-2021-08-029-024). This research was registered at the Clinical Research Information Service of the Republic of Korea (KCT0006751).

### 2.2 Data collection and baseline assessments

All participants underwent baseline assessments, including medical history and related medication, metabolic indices, and anthropometric measurements. BMI was calculated as weight (kg)/height (m^2^). Metabolic indices, such as waist circumference, blood pressure, heart rate, hemoglobin A1c, fasting blood glucose, and lipid profiles (total, low-density lipoprotein, and high-density lipoprotein cholesterol, and triglycerides) were measured.

### 2.3 Gastric sample collection

Four gastric mucosal tissue specimens were obtained under fasting conditions from the greater curvature of the antrum, using sterile biopsy forceps during endoscopic examination. Samples were collected under sterile conditions and stored in a refrigerator at −80 °C until DNA extraction. Rapid urease tests were performed to confirm the absence of *H. pylori* infection, and histological examinations ruled out atrophic gastritis and intestinal metaplasia.

### 2.4 16S rRNA sequencing of gastric microbiota

The gastric mucosal tissue specimens were subjected to 16S rRNA sequencing by Macrogen (Seoul, South Korea). Genomic DNA was extracted from the sample using a DNeasy PowerSoil Pro Kit (Qiagen, Hilden, Germany). We targeted the V3–V4 regions of the 16S rRNA gene within the genomic DNA by conducting polymerase chain reaction using two primers: 341F (5′-CCTACGGGNGGCWGCAG-3′) and 805R (5′-GACTACHVGGGTATCTAATCC-3′). The obtained amplicons were further processed according to the Illumina protocols to construct a sequencing library, which was then applied to the Illumina MiSeq platform to obtain paired-end (2×300 bp) reads. A representative library quality control report confirming the expected size distribution of the V3–V4 amplicons is provided in **Supplementary Figure S1**.

### 2.5 Amplicon sequence variant clustering of sequenced reads

All bioinformatic processes and analyses described below were performed within the Macrogen bioinformatics cloud platform. The raw reads were demultiplexed based on index sequences, and Cutadapt (v3.2) (Martin, 2011) was used to remove sequencing adapter and forward/reverse primer sequences. Subsequently, the reads were trimmed to obtain the forward (250 bp) and reverse (200 bp) sequence for paired-end reads. The trimmed reads were then error-corrected and denoised using the DADA2 (v1.18.0) (Callahan et al., 2016) package in R (v4.0.3). Reads with expected errors of 2 or more were removed. The sequencing-error-corrected paired-end reads were aligned with single sequences and chimeric sequences were eliminated using the consensus method in DADA2 to generate ASVs.

### 2.6 Analysis of microbial community

Each ASV sequence was subjected to BLAST+ (v2.9.0) (Camacho et al., 2009) against the NCBI *16S* Microbial Database to assign taxonomy information based on the most similar organism: if the best-hit query coverage was 85% or higher and the identity of the matched region was 85% or higher. To obtain a phylogenetic tree required for diversity analysis, ASVs were aligned using MAFFT (v7.475) (Katoh and Standley, 2013) and a phylogenetic tree was constructed from the alignment using FastTreeMP (v2.1.10) (Price et al., 2010). QIIME (v1.9) (Caporaso et al., 2010) was used for diversity analyses and data normalization through subsampling. To assess species diversity and evenness within samples, Shannon, Gini–Simpson, and phylogenetic diversity (PD) whole tree indices, were calculated and the relevance of alpha diversity was examined using rarefaction curves. Microbial community diversity among samples was determined based on Bray–Curtis and weighted/unweighted UniFrac distances. The relationships between samples were visualized using principal coordinates analysis (PCoA) and UPGMA trees (Rambault; Caporaso et al., 2010).

### 2.7 Metagenomic prediction of microbial community

To predict the MetaCyc metabolic pathways of microbial communities for each sample, Phylogenetic Investigation of Communities by Reconstruction of Unobserved States (PICRUSt2) (Douglas et al., 2020) was used. ASVs with a nearest sequenced taxon index value of ≥2 were excluded from the analysis. Heatmaps for visualization of selected pathways were designed using R (v4.4.1).

### 2.8 Statistical analysis of microbial communities

The Kruskal–Wallis test was used for comparisons among three groups, whereas the Wilcoxon rank-sum test was used for comparisons between two groups (Hollander et al., 2013). To compare beta diversity between groups, the previously calculated Bray–Curtis and weighted/unweighted UniFrac distance matrices were used for analysis of similarities (ANOSIM) (Clarke, 1993) and permutational multivariate analysis of variance (PERMANOVA) (Anderson, 2001). To compare the microbial community composition (relative abundance) between groups, linear discriminant analysis effect size (LEfSe) was used (Segata et al., 2011). Taxa with a linear discriminant analysis score of ≥2.0 and *P*-value ≤0.05 were selected (Hollander et al., 2013). The extent of differences was represented by the linear discriminant analysis score. The distribution of each taxon and significantly different microbial taxa identified using LEfSe were visualized using GraPhlAn (Asnicar et al., 2015). Spearman’s correlation (Best and Roberts, 1975) was calculated using species-level relative abundance and BMI variables of each sample. The correlation coefficient (Rho) and *P*-value for each species were calculated and species with a *P*-value ≤0.05 were visualized using scatter plots generated using ggplot (v3.5.1) (Wickham and Wickham, 2016) in R (v4.2.1).

## 3 Results

### 3.1 Baseline characteristics

The study included 30 participants, who were divided into three groups based on their BMI: normal weight (18.5–22.9 kg/m²), overweight (23.0–24.9 kg/m²), and obese (≥25.0 kg/m²). Although not statistically significant due to the small number of participants, there was a lower proportion of males in the normal weight group compared with that in the other groups. The body weight showed a significant difference among the three groups: 55.8 ± 3.7 kg in the normal weight group, 69.3 ± 6.2 kg in the overweight group, and 73.2 ± 11.6 kg in the obese group (*P*<0.001; **Table 1**). Similarly, the waist circumference was 75.4 ± 4.0, 86.7 ± 7.4, and 91.4 ± 7.1 cm in the normal weight, overweight, and obese groups, respectively (*P*<0.001). The hip circumference also showed a significant difference, measuring 90.0 ± 1.9, 97.2 ± 6.0, and 99.3 ± 4.2 cm in the three groups (*P*<0.001). Regarding underlying medical conditions, there were no significant differences among the groups. Hemoglobin levels were significantly lower in the normal weight group (12.5 ± 1.8 g/dL) compared with that in the overweight (14.5 ± 2.8 g/dL) and obese (14.5 ± 1.7 g/dL) groups (*P*=0.021). Other laboratory parameters showed no significant differences between the groups.

**Table 1:** Demographic and clinical characteristics of study population.

Variables	Normal weight	Overweight	Obesity	*P*-value (F, df)
	(*n*=10)	(*n*=10)	(*n*=10)	
Sex, Male (%)	2 (20%)	6 (60%)	5 (50%)	0.182 (3.412, 2)
Age (year)	50.1 ± 7.9	48.4 ± 13.7	49.6 ± 11.4	0.993 (0.014, 2)
Height (cm)	160.8 ± 5.9	170.0 ± 8.05	164.3 ± 9.2	0.075 (5.182, 2)
Weight (cm)	55.8 ± 3.7	69.3 ± 6.2	73.2 ± 11.6	<.001 (16.733, 2)
Waist circumference (cm)	75.4 ± 4.0	86.7 ± 7.4	91.4 ± 7.1	<.001 (15.704, 2)
Hip circumference (cm)	90.0 ± 1.9	97.2 ± 6.0	99.3 ± 4.2	<.001 (13.735, 2)
Underlying disease				
Diabetes mellitus	0 (0%)	0 (0%)	0 (0%)	1.000 (0.000, 2)
Thyroid disease	0 (0%)	0 (0%)	0 (0%)	1.000 (0.000, 2)
Hypertension	0 (0%)	1 (10%)	0 (0%)	0.368 (2.000, 2)
Cerebrovascular disease	0 (0%)	0 (0%)	0 (0%)	1.000 (0.000, 2)
Tuberculosis	0 (0%)	0 (0%)	0 (0%)	1.000 (0.000, 2)
Liver disease	0 (0%)	0 (0%)	0 (0%)	1.000 (0.000, 2)
Renal disease	0 (0%)	0 (0%)	0 (0%)	1.000 (0.000, 2)
Hyperlipidemia	0 (0%)	2 (20%)	1 (10%)	0.342 (2.148, 2)
Surgical history	0 (0%)	2 (20%)	1 (10%)	0.342 (2.148, 2)
Drug history				
Probiotics	0 (0%)	0 (0%)	0 (0%)	1.000 (0.000, 2)
Nonsteroidal anti-inflammatory drugs	0 (0%)	0 (0%)	0 (0%)	1.000 (0.000, 2)
Aspirin	0 (0%)	0 (0%)	0 (0%)	1.000 (0.000, 2)
Steroid	0 (0%)	0 (0%)	0 (0%)	1.000 (0.000, 2)
Anticoagulants	0 (0%)	1 (10%)	0 (0%)	0.368 (2.000, 2)
Smoking	0 (0%)	1 (10%)	2 (20%)	0.342 (2.148, 2)
Alcohol	1 (10%)	4 (40%)	1 (10%)	0.163 (3.625, 2)
Vital sign				
Systolic blood pressure (mmHg)	118 ± 14.6	126.0 ± 11.6	131.0 ± 11.1	0.071 (5.280, 2)
Diastolic blood pressure (mmHg)	77.5 ± 8.3	77.8 ± 11.2	78.0 ± 5.7	0.940 (0.124, 2)
Heart rate	78.9 ± 10.4	77.9 ± 12.1	79.8 ± 13.5	0.898 (0.214, 2)
Laboratory findings				
Whole blood count	5,320.0 ± 1,291.7	5,260.0 ± 1,082.4	6,328.0 ± 1,486.8	0.218 (3.050, 2)
Hemoglobin	12.5 ± 1.8	14.5 ± 2.8	14.5 ± 1.7	0.021 (7.683 2)
Platelet count	266,928.1 ± 111,359.8	260,500.0 ± 91,147.3	237,723.2 ± 91,371.0	0.453 (1.583, 2)
Blood urea nitrogen	11.9 ± 3.1	13.9 ± 3.1	13.3 ± 3.9	0.302 (2.395, 2)
Creatine	0.7 ± 0.2	0.8 ± 0.2	0.8 ± 0.2	0.366 (2.010, 2)
Total bilirubin	0.8 ± 0.3	0.9 ± 0.4	0.704 ± 0.209	0.581 (1.087, 2)
Aspartate aminotransferase	25.2 ± 5.1	29.5 ± 9.5	30.7 ± 15.1	0.636 (0.905, 2)
Alanine aminotransferase	22.1 ± 9.0	31.3 ± 15.1	35.1 + 22.86	0.173 (3.504, 2)
Gamma glutamic pyruvate transaminase	18.0 ± 6.1	28.6 ± 15.8	30.9 + 20.55	0.096 (4.697, 2)
Fasting blood glucose	94.0 + 11.29	98.1 + 4.30	103.4 ± 6.9	0.121 (4.220, 2)
Hemoglobin A1c	5.7 ± 0.2	5.5 ± 0.3	5.6 ± 0.3	0.348 (2.111, 2)
Total cholesterol	204.0 ± 29.5	207.5 ± 30.3	214.9 + 42.76	0.941 (0.122, 2)
Triglycerides	69.7 ± 28.3	117.6 ± 67.5	117.6 + 40.738	0.009 (9.425, 2)
High density lipoprotein	62.2 ± 12.7	56.0 ± 14.6	49.6 ± 9.8	0.111 (4.396, 2)
Low density lipoprotein	121.8 ± 21.7	120.8 ± 32.3	134.3 ± 37.6	0.717 (0.667, 2)

Data are expressed as mean ± standard deviation (SD) or number (%).

df, degree of freedom.

### 3.2 Statistics of sequenced reads

We obtained raw reads (104,637 ± 36,054 reads/sample) from the Illumina sequencing. After filtering (see Materials and Methods), a total of 46,685 ± 19,672 reads/sample were retained for ASV determination. The read count per sample ranged from 13,886 to 122,502. From these reads, a total of 2,985 ASV sequences were assembled. These results indicate that our dataset was sufficient for subsequent microbial community analysis.

### 3.3 Alpha diversity of gastric microbiota

**Figure 1:**
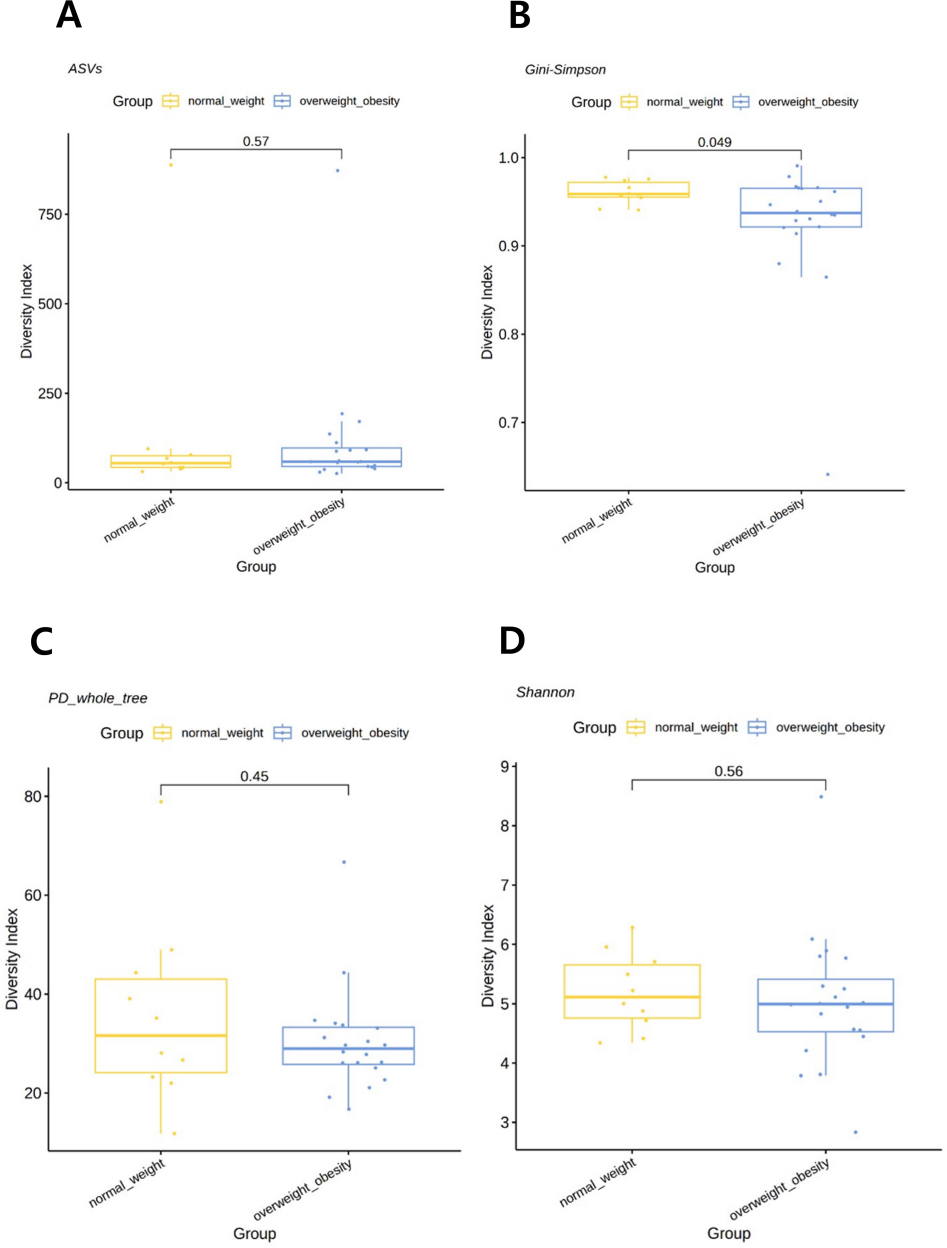
Comparison of alpha diversity between the overweight/obese and normal weight groups. **(A)** Observed amplicon sequence variants (ASVs). **(B)** Gini–Simpson index. **(C)** Phylogenetic diversity (PD) whole tree. **(D)** Shannon index. *P*-values were determined using the Wilcoxon rank-sum test.

When the participants were categorized into two groups—overweight/obese and normal weight—alpha diversity, as assessed using the Gini–Simpson index for evenness, was significantly lower in the overweight/obese group than that in the normal weight group (*P*=0.049). However, the PD whole tree index (*P*=0.4480), Shannon index (*P*=0.5588), or observed ASVs (*P*=0.5672) were not statistically different among groups (**Figure 1**). When analyzed as three separate BMI groups, a trend of reduced diversity in overweight and obese individuals was observed, although not statistically significant (see **Supplementary Figure S2**).

### 3.4 Beta diversity of gastric microbiota

**Figure 2:**
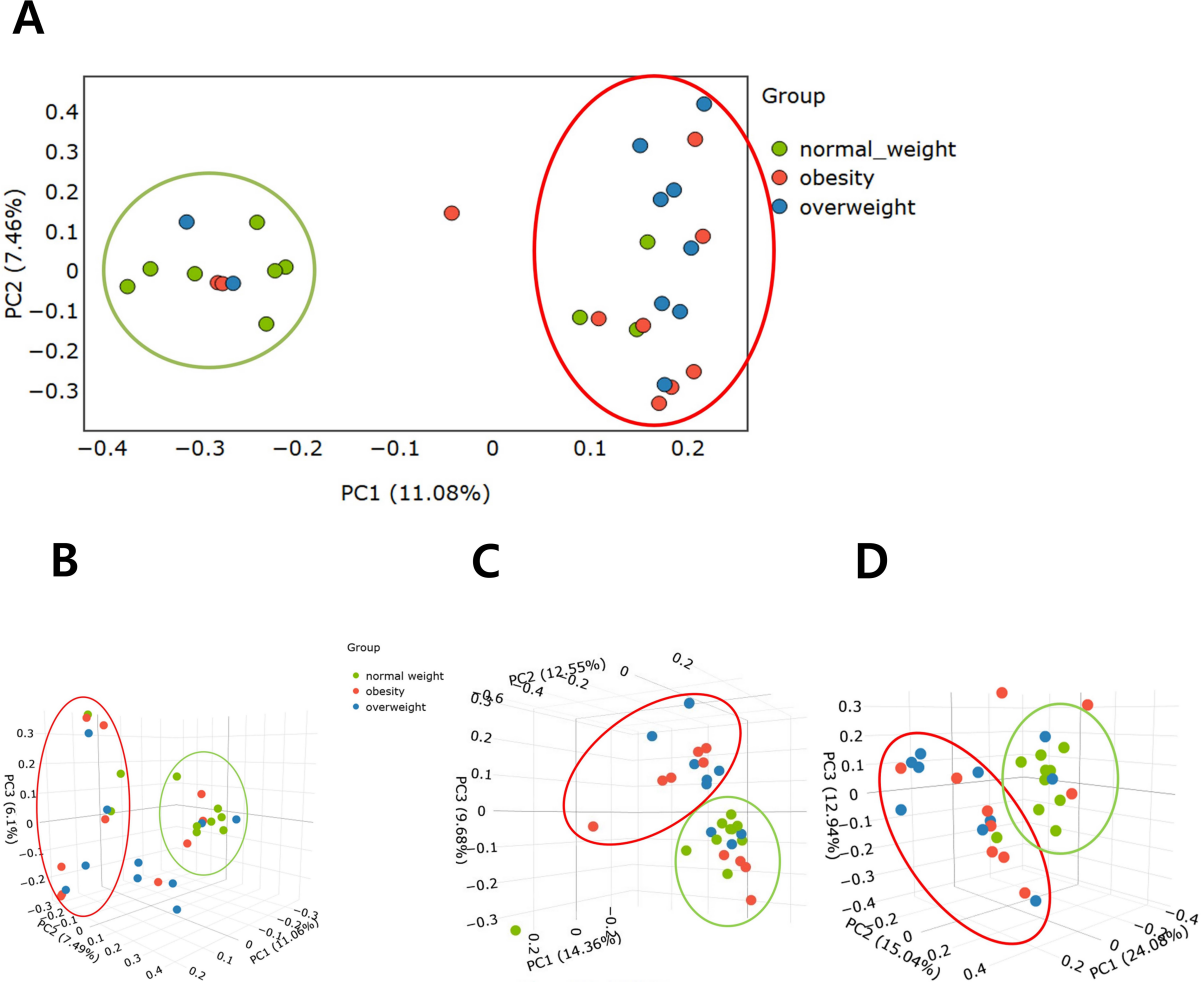
Comparison of beta diversity among the overweight, obese, and normal weight groups. **(A)** Bray–Curtis distance (2D). **(B)** Bray–Curtis distance (3D). **(C)** Unweighted UniFrac distance matrix. **(D)** Weighted UniFrac distance matrix.

PCoA demonstrated clear separations in microbial composition among the three BMI groups, indicating significant differences in beta diversity. Statistical analyses showed that Bray–Curtis distances assessed by ANOSIM revealed significant differences among the three groups (*P*=0.005) (**Figures 2A, B**), as did unweighted UniFrac distances tested by PERMANOVA (*P*=0.004) (**Figure 2C**). Pairwise comparisons showed significant differences between the normal weight and overweight groups (*P*=0.02 and 0.005, respectively), and between the normal weight and obese groups (*P*=0.05 and 0.035, respectively). However, no significant differences were observed between the overweight and obese groups. In addition, weighted UniFrac distance, also assessed by PERMANOVA, demonstrated a significant difference between the normal weight and overweight groups (*P*=0.02) (**Figure 2D**).

### 3.5 Composition of gastric microbiota

**Figure 3:**
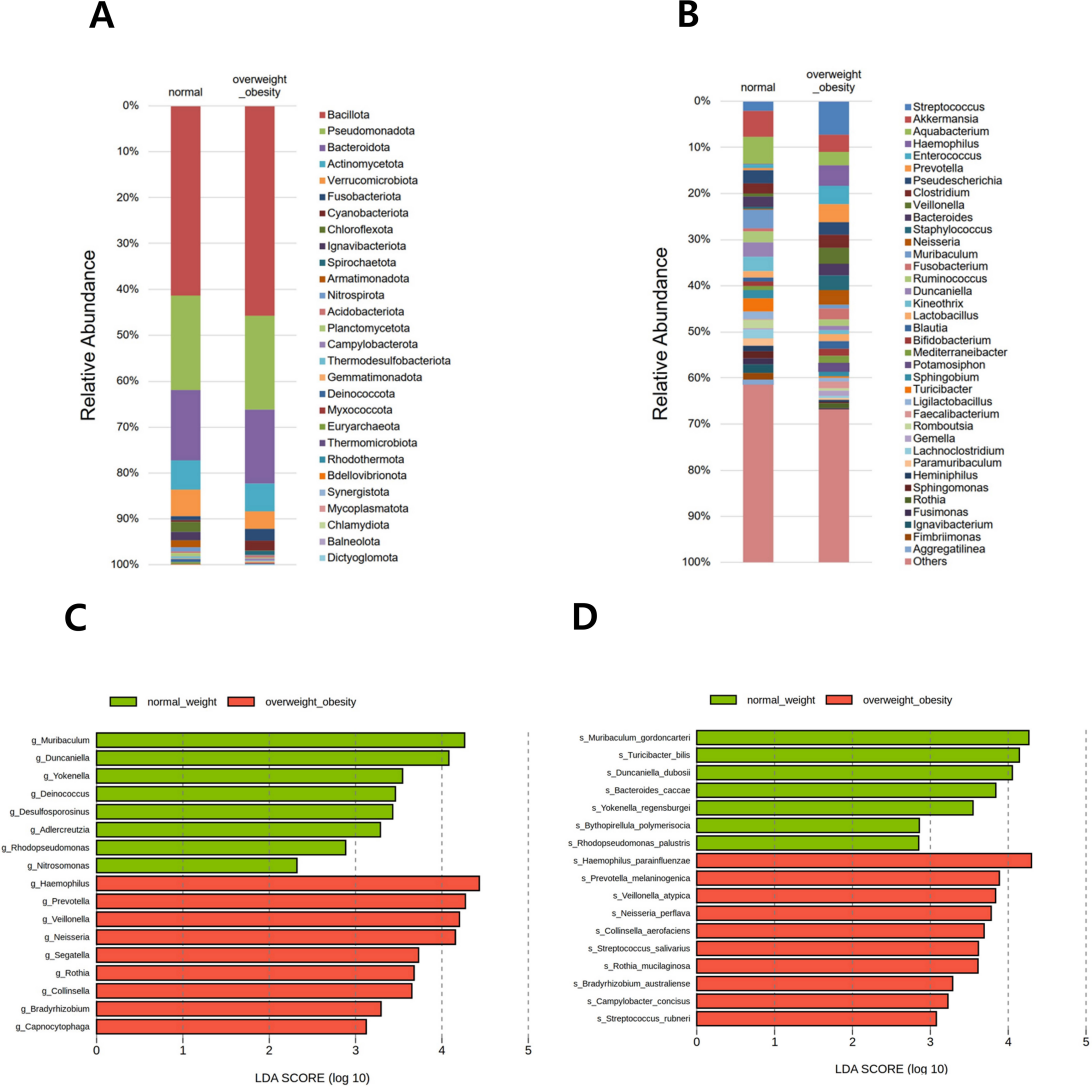
Relative abundance of the microbial community and differentially abundant taxa. Stacked bar plots show the taxonomic composition at the **(A)** phylum and **(B)** genus levels. All detected phyla are included, whereas genera are presented if the relative abundance in any group exceeded 1.0%. Differentially abundant taxa between overweight/obese and normal-weight groups were identified using linear discriminant analysis effect size (LEfSe) at the **(C)** genus and **(D)** species levels.

In total, 28 phyla, 653 genera, and 1,118 species were identified across all samples. Relative abundance was visualized using two stacked bar plots at the phylum and genus levels (**Figures 3A, B**). The six predominant phyla—Bacillota, Pseudomonadota, Bacteroidota, Actinomycetota, Verrucomicrobiota, and Fusobacteriota—accounted for 93.3% of the gastric microbiota. At the genus level, *Streptococcus*, *Akkermansia*, *Aquabacterium*, *Haemophilus*, *Enterococcus*, *Prevotella*, *Pseudescherichia*, *Clostridium*, *Veillonella*, *Bacteroides*, *Staphylococcus*, *Neisseria*, *Muribaculum*, and *Fusobacterium* collectively comprised 40.0% of the total microbial composition.

In the comparison between the overweight/obese and normal weight groups, LEfSe analysis at the genus level identified 17 genera with significant differences. Among them, *Muribaculum*, *Duncaniella*, *Yokenella*, *Deinococcus*, *Desulfosporosinus*, *Adlercreutzia*, *Rhodopseudomonas*, and *Nitrosomonas* were reduced in the overweight/obese group compared with those in the normal weight group. Conversely, *Haemophilus*, *Prevotella*, *Veillonella*, *Neisseria*, *Segatella*, *Rothia*, *Collinsella*, *Bradyrhizobium*, and *Capnocytophaga* were increased in the overweight/obese group (**Figure 3C**). In LEfSe analysis at the species level, 17 bacterial species were significantly different between the BMI groups. Notably, *Muribaculum gordoncarteri, Turicibacter bilis, Duncaniella dubosii, Bacteroides caccae, Yokenellar regensburgei, Bythopirellula polymerisocia*, and *Rhodopseudomonas palustris* were significantly reduced in the overweight/obese group compared with those in the normal weight group. Conversely, other species, such as *Haemophilus parainfluenzae, Prevotella melaninogenica, Veillonella atypica, Neisseria perflava, Collinsella aerofaciens, Streptococcus salivarius, Rothia mucilaginosa, Bradyrhizobium australiense, Campylobacter concisus*, and *Streptococcus rubneri* showed increased abundance in the overweight/obese group (**Figure 3D**).

In the three-group comparison among the normal weight, overweight, and obese groups at the genus level, *Muribaculum*, *Yokenella*, and *Rhodopseudomonas* abundance were higher in the normal weight group, whereas *Haemophilus*, *Prevotella*, and *Neisseria* abundance were higher in the overweight group (see **Supplementary Figure S3**). Additionally, *Segatella* and an unclassified genus were more abundant in the obese group. At the species level, *M. gordoncarteri*, *Bacteroides caccae*, and *Y. regensburgei* were more abundant in the normal weight group, whereas *H. parainfluenzae*, *N. perflava*, and *Haemophilus pittmaniae* were more abundant in the overweight group. Additionally, *Ruminococcus gauvreauii* and *Clostridium innocuum* were more abundant in the obese group.

### 3.6 Functional profiling of gastric microbiota

**Figure 4:**
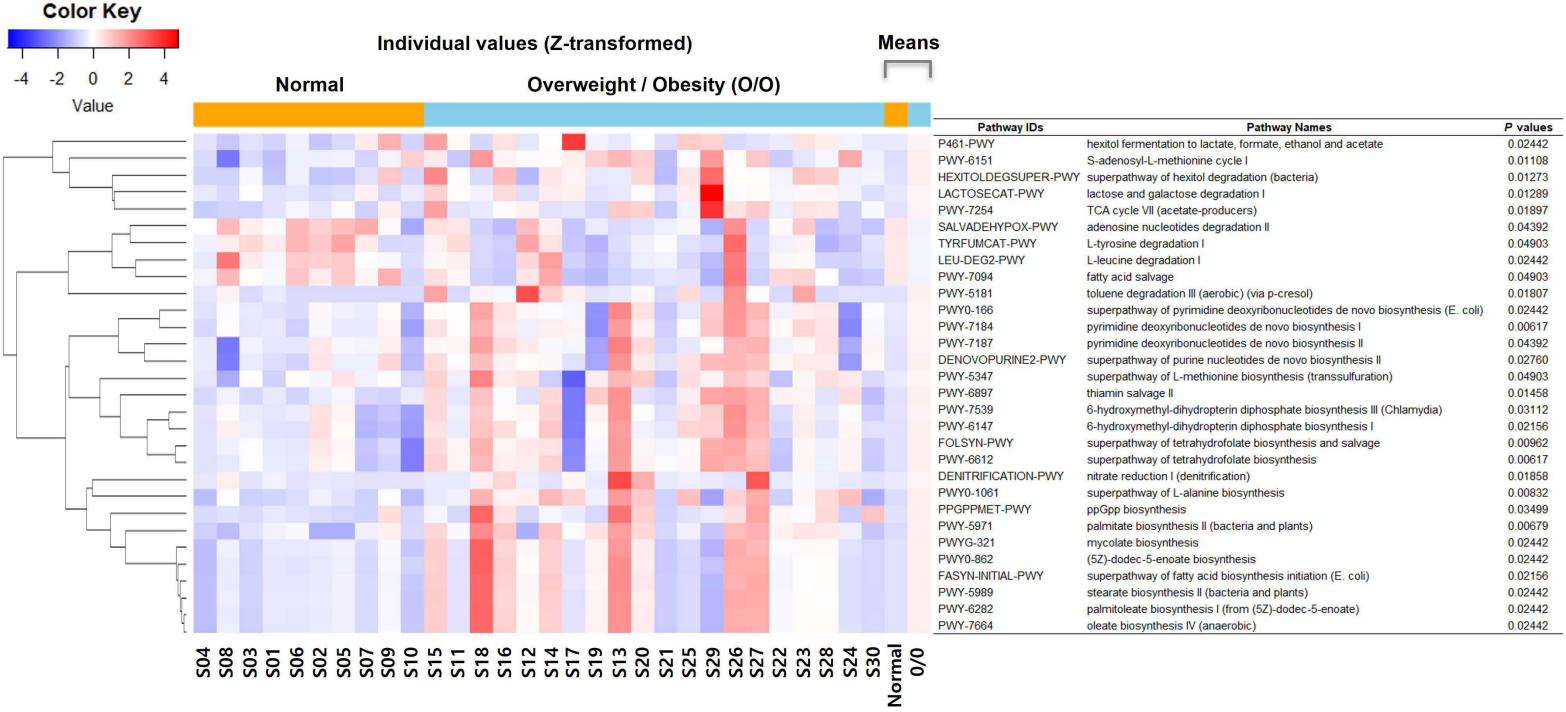
Functional prediction analysis using phylogenetic investigation of communities by reconstruction of unobserved states (PICRUSt2) between the overweight/obese and normal weight groups. TCA, tricarboxylic acid; *E. coli*, *Escherichia coli*.

Functional prediction analysis using PICRUSt2 between the two groups (overweight/obese *vs.* normal weight) revealed that metabolic pathways related to fatty acid synthesis, amino acid synthesis/degradation, vitamin synthesis, S-adenocyl-L-methionine (SAM) synthesis, carbohydrate metabolism, and mycolate synthesis differed across BMI categories (**Figure 4**).

Fatty acid biosynthesis pathways, including palmitate biosynthesis II (bacteria and plants), superpathway of fatty acid biosynthesis initiation (*Escherichia coli*), palmitoleate biosynthesis I (from (5Z)-dodec-5-enoate), (5Z)-dodec-5-enoate biosynthesis, stearate biosynthesis II (bacteria and plants), and oleate biosynthesis IV (anaerobic), were significantly associated with the overweight/obese group. In contrast, the pathway related to fatty acid salvage was significantly upregulated in the normal weight group.

Amino acid synthesis pathways, such as the superpathway of L-alanine biosynthesis and L-methionine biosynthesis (transsulfuration), were significantly associated with the overweight/obese group. Amino acid degradation pathways, such as L-leucine degradation I and L-tyrosine degradation I were downregulated in the overweight/obese group.

Vitamin synthesis pathways, including superpathway of tetrahydrofolate biosynthesis, superpathway of tetrahydrofolate biosynthesis and salvage, thiamin salvage II, 6-hydroxymethyl-dihydropterin diphosphate biosynthesis I, and 6-hydroxymethyl-dihydropterin diphosphate biosynthesis III (*Chlamydia*) showed high activity in the overweight/obese group.

SAM synthesis pathways, such as SAM cycle I, superpathway of tetrahydrofolate biosynthesis, superpathway of tetrahydrofolate biosynthesis and salvage, and superpathway of L-methionine biosynthesis (transsulfuration) were significantly associated with the overweight/obese group.

Carbohydrate metabolism pathways, such as TCA cycle VII (acetate-producers), lactose and galactose degradation I, superpathway of hexitol degradation (bacteria), and hexitol fermentation to lactate, formate, ethanol, and acetate were significantly associated with the overweight/obese group.

The mycolate biosynthesis pathway was significantly high in the overweight/obese group.

### 3.7 Correlation between BMI and gastric microbiota

Correlation analysis further emphasized the associations between specific bacterial species and BMI. Reduced abundance of *M. gordoncarteri* (Rho=−0.4, *P*=0.027), *T. bilis* (Rho=−0.4, *P*=0.0286), *D. dubosii* (Rho=−0.37, *P*=0.0426), *Aquabacterium commune* (Rho=−0.36, *P*=0.0487)*, Bifidobacterium pseudolongum* (Rho=−0.39, *P*=0.034), and *Sphingobium xenophagum* (Rho=−0.39, *P*=0.034) negatively correlated with BMI, suggesting a potential role in obesity-related metabolic dysregulation (see **Supplementary Figure S4**).

## 4 Discussion

This study demonstrates significant differences in the composition and functional profiles of the gastric microbiota between BMI categories, suggesting gastric microbial communities, like their intestinal counterparts, may play a role in obesity-related metabolic alterations (Ley et al., 2005; Turnbaugh et al., 2009; Denou et al., 2016; Yun et al., 2017; Kim et al., 2020; Duan et al., 2021).

In this study, among the four indices related to alpha diversity, only the Gini–Simpson index for evenness significantly decreased in overweight and obese individuals. This aligns with previous studies on the intestinal microbiota, where dysbiosis and decreased microbial diversity were strongly associated with obesity and metabolic disorders (Turnbaugh et al., 2009; Denou et al., 2016; Yun et al., 2017; Kim et al., 2020; Duan et al., 2021). This reduced diversity may impair the microbiota functional capacity, including energy homeostasis and anti-inflammatory processes, as shown in other regions of the gastrointestinal tract.

Although some studies have reported no significant differences, obesity has generally been associated with changes in the composition of the gut microbiota, including an increased relative abundance of Bacillota at the phylum level and a higher Bacillota/Bacteroidota ratio compared to individuals with normal weight (Ley et al., 2005; Andoh et al., 2016; Koliada et al., 2017; Murga-Garrido et al., 2022). In this study, there was no significant difference in the gastric microbiota at the phylum level among normal weight, overweight, and obese groups. Seventeen bacterial species showed significant differences between the normal weight and overweight/obese groups. *M. gordoncarteri*, *T. bilis*, and *D. dubosii* were notably reduced in the overweight/obese group and are linked to lipid and carbohydrate metabolism (Lagkouvardos et al., 2019; Chung et al., 2020; Zhu et al., 2024), whereas *H. parainfluenzae* and *V. atypica* were more abundant and may contribute to metabolic dysregulation via pro-inflammatory pathways. A meta-analysis identified Pseudomonadota (formerly Proteobacteria) as the phylum most consistently associated with obesity (Xu et al., 2022). Several species belonging to Pseudomonadota, such as *Proteus mirabilis* and *E. coli*, promote gastrointestinal inflammation, contributing to insulin resistance and metabolic diseases (Longstreth, 2007; Zhang et al., 2021). In this study, *H. parainfluenzae*, *N. perflava*, *B. australiense*, and *C. concisus*—all within this phylum—were more abundant in the overweight/obese group.

The obesity-related microbes identified in previous studies using fecal samples, such as *H. parainfluenzae*, *V. atypica*, and *R. mucilaginosa* were also found in this study in gastric mucosal samples. This suggests that changes in these microbes may originate in the stomach, leading to their increase or decrease throughout the gastrointestinal tract, ultimately affecting the colon and fecal microbiota. Notably, the altered abundance of *D. dubosii*, *B. polymerisocia*, and *R. palustris* (decreased), and *N. perflava*, *B. australiense*, *C. concisus*, *S. rubneri* (increased) in the overweight/obese group has not been previously reported in studies using intestinal or fecal microbiota. These shifts may reflect unique gastric environmental factors rather than obesity per se. Given that *H. pylori* infection alters gastric microbial diversity during progression to atrophic gastritis and intestinal metaplasia (Noto and Peek, 2017), we excluded individuals with such conditions to minimize confounding by inflammation. Therefore, the observed changes likely reflect gastric-specific rather than inflammation-driven microbiota alterations.

The gut microbiota contribute to obesity via pathways involving energy harvest, inflammation, and lipid and bile acid metabolism (Kim, 2023; Zhuang et al., 2023). Gut microbiota play a crucial role in the host’s energy harvest, as demonstrated in germ-free mouse models where mice harboring gut microbiota exhibited greater weight gain and higher energy absorption despite consuming the same diet (Lupp et al., 2012; Rooks and Garrett, 2016). In this study, PICRUSt2 analysis revealed BMI-related differences in fatty acid, amino acid, and vitamin metabolism. The increased activity of fatty acid biosynthesis pathways in obesity aligns with prior findings linking this shift to enhanced energy extraction and adipogenesis​​ (Turnbaugh et al., 2006; Kim et al., 2020; Duan et al., 2021). Furthermore, the diminished activity of amino acid degradation pathways in obese individuals may suggest alterations in protein metabolism. Although short-chain fatty acids (SCFAs) support lipid metabolism and intestinal health, their role in obesity remains debated due to variable effects (Turnbaugh et al., 2006; Chambers et al., 2015). In addition, gut microbes modulate bile acid deconjugation, which influences hepatic cholesterol and lipid metabolism (Kalaany and Mangelsdorf, 2006; Watanabe et al., 2006; Wahlström et al., 2016). While we identified alterations in predicted pathways related to amino acid metabolism (e.g., degradation) and SAM biosynthesis using PICRUSt2, our study did not include untargeted metabolomics or direct quantification of SAM/S-adenosylhomocysteine levels. Although microbial functions can be inferred using PICRUSt2, the lack of supporting data such as serum metabolite measurements or untargeted metabolomics prevents confirmation of the metabolic relevance of the gastric microbiota in this study.

Among the species significantly associated with BMI, there is limited research on the association between *M. gordoncarteri* and metabolic diseases. However, the *Muribaculum* genus plays a key role in carbohydrate metabolism, particularly glycolysis and gluconeogenesis (Chung et al., 2020; Zhu et al., 2024), which are central to energy regulation and may influence obesity. *Muribaculum* also contributes to SCFA production from dietary fiber, supporting energy balance, lipid metabolism, and anti-inflammatory effects beneficial in metabolic disorders like obesity and type 2 diabetes. Additionally, through bile salt hydrolase activity, *Muribaculum* affects bile acid metabolism. Its depletion has been associated with reduced levels of hyodeoxycholic and ursodeoxycholic acid, bile acids that activate the intestinal farnesoid X receptor (FXR), thereby influencing lipid absorption and bile acid synthesis via the FXR– fibroblast growth factor 19 axis (Xu et al., 2023). Therefore, reduced *Muribaculum* abundance may contribute to lipid dysregulation through impaired bile acid signaling.

Although *D. dubosii* has not been directly linked to obesity or metabolic diseases in humans or animal models, it belongs to the *Muribaculaceae* family, known for its role in carbohydrate metabolism and SCFA production—key processes in energy and lipid regulation (Lagkouvardos et al., 2019; Chung et al., 2020). Given its reduced abundance in obese individuals, further research is warranted to clarify its potential involvement in metabolic dysregulation via impaired carbohydrate metabolism and SCFA-associated protective functions.

Studies suggest that *Turicibacter* genus, including *T. bilis*, may affect host lipid metabolism and be associated with obesity-related changes in body weight (Fung et al., 2019; Lynch et al., 2023). A systematic review also reported lower levels of *Turicibacter*-related taxa in obese individuals compared to lean controls, indicating a potential protective role against metabolic dysfunction (Xu et al., 2022).

Regarding chronic inflammation, lipopolysaccharides from gram-negative bacteria can cross the intestinal barrier and trigger systemic inflammation via the Toll-like receptor-4 pathway, promoting the production of pro-inflammatory cytokines (such as IL-1, IL-6, and TNF-α) and contributing to insulin resistance (Cani et al., 2009; Lee et al., 2020). The elevated abundance of *H. parainfluenzae* and *V. atypica* in obese individuals in our study suggests a possible role of gastric microbiota in systemic inflammation and metabolic disorders. *H. parainfluenzae* is known to elicit mucosal immune responses in chronic obstructive pulmonary disease (Mitchell and Hill, 2000), while *V. atypica*, prevalent in dysbiotic oral biofilms, has been linked to altered inflammatory and nutritional status in children (Theodorea et al., 2022).

Several studies have linked *H. pylori* infection to metabolic dysregulation, including obesity and insulin resistance (Azami et al., 2021; Baryshnikova et al., 2024). For instance, Azami et al. (2021) reported significantly elevated BMI and insulin resistance in *H. pylori*-positive individuals, while Baryshnikova et al. (2024) suggested that reduced ghrelin levels and chronic inflammation may underlie these metabolic disturbances. To avoid confounding effects of infection-induced microbiota shifts, our study excluded *H. pylori*-positive subjects, allowing for a more accurate assessment of BMI-associated changes in gastric microbiota among non-infected individuals. Future research directly comparing *H. pylori*-positive and -negative populations across BMI categories could provide valuable insights into the interactive effects of *H. pylori* infection and host metabolic status on gastric microbial ecology.

The association between gastric microbiota and BMI highlights the stomach as a potential therapeutic target for modulating obesity. Interventions targeting the gastric microbiota, such as probiotics or dietary modifications, may complement existing treatments aimed at restoring gut microbial balance. Additionally, the distinct microbial profiles observed in this study warrant further research to elucidate the causal relationships between specific bacterial taxa and metabolic pathways.

This study had several limitations. First, the small sample size (n=10 per group) substantially limits the statistical power and generalizability of our findings. In particular, the limited sample size increases the risk of false-positive or -negative results and precludes robust detection of subtle microbial differences. Furthermore, the unequal sex distribution in the normal weight group and the absence of sex-stratified statistical analysis limits our ability to draw definitive conclusions regarding sex-specific microbial patterns. In our study, the proportion of women was higher than that of men in the normal weight group. Subgroup analysis based on sex (**Supplementary Figure S5A**) revealed that female participants exhibited lower alpha diversity metrics—including the Shannon index, observed ASVs, and Faith’s PD—compared to their male counterparts; however, these differences were not statistically significant (*P*>0.05). Furthermore, we confirmed that the separation observed between the normal and overweight/obese groups in the beta diversity of PCoA (**Figure 2A**) may not have been influenced by this sex imbalance (**Supplementary Figure S5B**). In addition, most participants in this study resided in a specific region of South Korea, which limits the ability to reflect geographic diversity related to dietary habits and lifestyle. Given the homogeneity of the study population, further validation in multi-center and multi-ethnic cohorts is necessary to confirm the findings. Second, this study is a cross-sectional analysis comparing overweight/obese and normal-weight groups using 16S rRNA gene sequencing. Due to the nature of this design, it is difficult to determine whether the observed differences in microbial composition are a cause or consequence of obesity. Third, although we excluded individuals with *H. pylori* infection to minimize its confounding effect on the gastric microbiota, other potential confounders such as dietary habits, physical activity, medication use, socioeconomic status, and host genetic factors were not systematically assessed and thus could not be adjusted for. In addition, local physiological variables, including gastric pH and mucosal immune markers such as cytokine levels, were not measured. This was due to the limited volume of biopsy tissue obtained from clinical endoscopy. Nonetheless, these factors may be important in shaping the gastric microbial environment. Lastly, we acknowledge that deeper functional validation, such as shotgun metagenomics, metatranscriptomics, or experimental verification of microbial metabolic activity, was not performed. While our findings suggest that taxa such as *Muribaculum* and *Turicibacter* are linked to metabolic pathways involving SCFA production and bile acid metabolism, our study did not include direct measurement of host biomarkers such as circulating bile acids, inflammatory cytokines, or fecal SCFA concentrations. These data would be valuable in confirming the functional consequences of microbial shifts. Therefore, future studies with larger, geographically diverse cohorts and balanced sex distributions are necessary to validate these preliminary observations. Moreover, longitudinal studies integrating host metabolic profiles with microbial community data—using multi-omics approaches such as metabolomics, microbial genome analysis, and host genotyping—and accounting for dietary habits, local physiological parameters, and other potential confounding factors, would be valuable to better characterize the functional implications of gastric microbiota alterations in obesity.

In conclusion, our findings highlight significant differences in the composition and functional potential of the gastric microbiota across BMI categories. The identification of specific microbial taxa and metabolic pathways associated with obesity provides a valuable basis for future research into the metabolic roles of the gastric microbiota and its potential as a therapeutic target.

## Data Availability

The datasets presented in this study can be found in online repositories. The name of the repository and accession number can be found below: NCBI; PRJNA1270037.

## Supplementary material

Supplementary Figure 1 Representative library quality control reports showing fragment size distribution of V3–V4 amplicons, confirming successful amplification and appropriate library preparation for sequencing.

Supplementary Figure 2 Comparison of alpha diversity metrics of gastric microbiota among three BMI-defined groups (normal, overweight, obese). **(A)** Observed amplicon sequence variants (ASVs). **(B)** Gini–Simpson index. **(C)** Phylogenetic diversity (PD) whole tree. **(D)** Shannon index. *P*-values were determined using the Wilcoxon rank-sum test.

Supplementary Figure 3 Comparative analysis of gastric microbiota composition among normal weight, overweight, and obese groups. **(A)** Cladogram generated from linear discriminant analysis effect size (LEfSe) showing differentially abundant taxa across the three BMI groups (normal weight, overweight, and obese). **(B)** Histogram of the linear discriminant analysis (LDA) scores computed for features with differential abundance among the three BMI groups.

Supplementary Figure 4 Correlation analysis between specific bacterial species and body mass index. **(A)***Muribaculum gordoncarteri*, **(B)***Turicibacter bilis*, **(C)***Duncaniella dubosii*, **(D)***Aquabacterium commune*, **(E)***Bifidobacterium pseudolongum*, and **(F)***Sphingobium xenophagum*.

Supplementary Figure 5 Sex-based analysis of alpha and beta diversity in gastric microbiota. **(A)** Rarefaction curves of the Shannon index according to sex. **(B)** Principal coordinates analysis (PCoA) based on Bray–Curtis distance colored by sex.
