# Correction: Evaluation of the gastric microbiota based on body mass index using 16S rRNA gene sequencing

**DOI:** 10.3389/fcimb.2025.1702282

**Published:** 2025-10-24

**Authors:** Sang Hoon Lee, Eun Bae Kim, Sung Chul Park, Seung-Joo Nam, Hyunseok Cho, Han Jo Jeon, Sang Pyo Lee

**Affiliations:** ^1^ Department of Internal Medicine, Kangwon National University College of Medicine, Chuncheon, Republic of Korea; ^2^ Department of Applied Animal Science, Kangwon National University College of Animal Life Sciences, Chuncheon, Republic of Korea; ^3^ Institute of Animal Life Science, Kangwon National University, Chuncheon, Republic of Korea; ^4^ Department of Pediatrics, Kangwon National University College of Medicine, Chuncheon, Republic of Korea; ^5^ Department of Internal Medicine, Korea University College of Medicine, Seoul, Republic of Korea; ^6^ Department of Internal Medicine, Hanyang University College of Medicine, Seoul, Republic of Korea

**Keywords:** body mass index, gastric microbiota, obesity, 16S rRNA sequencing, metabolic dysregulation

In the published article, **Figures 2A–D** and **Figures 3A–D** were mistakenly interchanged. The corrected arrangement is: **Figure 2A–D** should appear as **Figure 3A–D** , and **Figure 3A–D** should appear as **Figure 2A–D** .

The original article has been updated.

**Figure 2:**
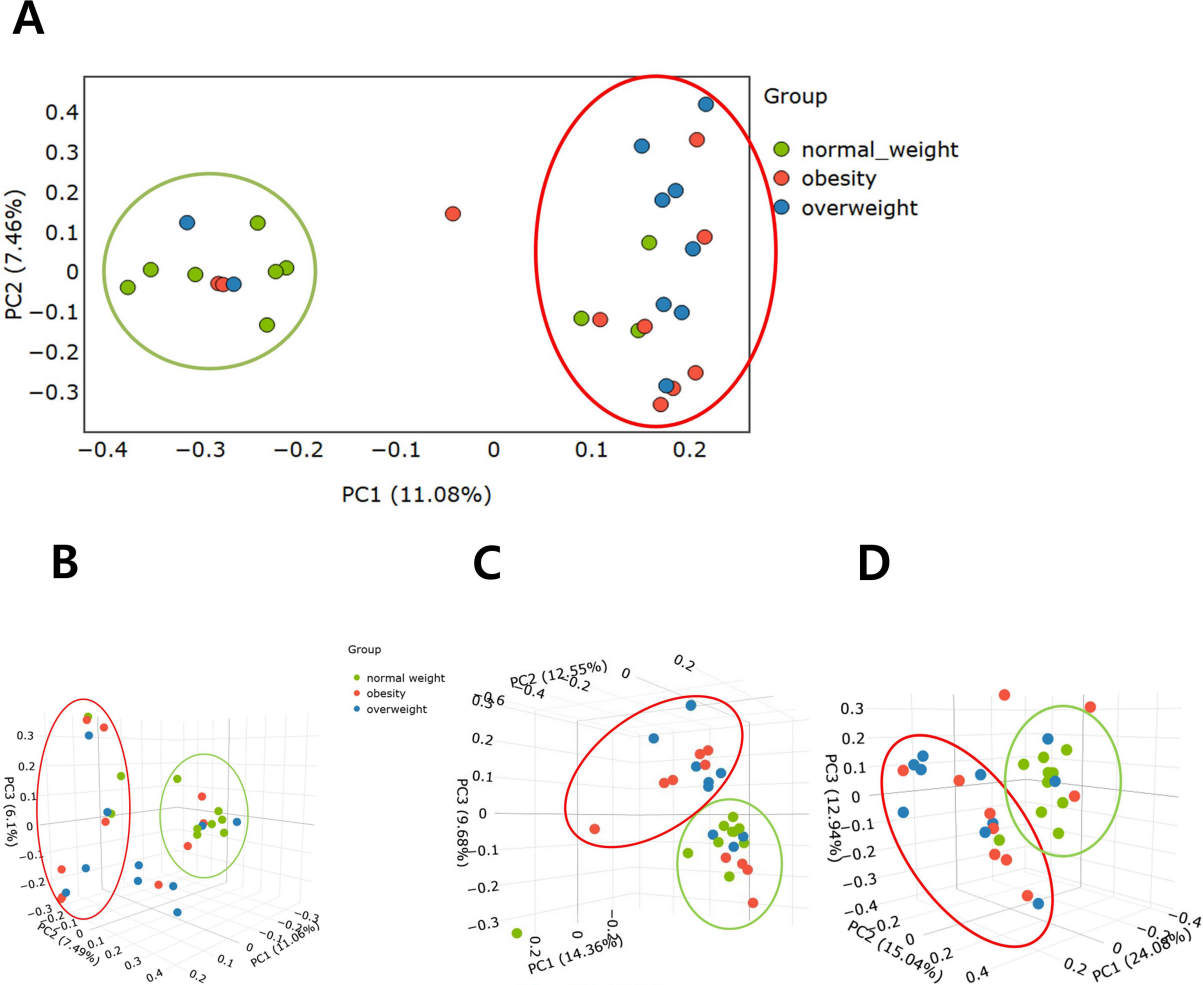
Comparison of beta diversity among the overweight, obese, and normal weight groups. **(A)** Bray -Curtis distance (2D). **(B)** Bray -Curtis distance (3D). **(C)** Unweighted UniFrac distance matrix. **(D)** Weighted UniFrac distance matrix..

**Figure 3:**
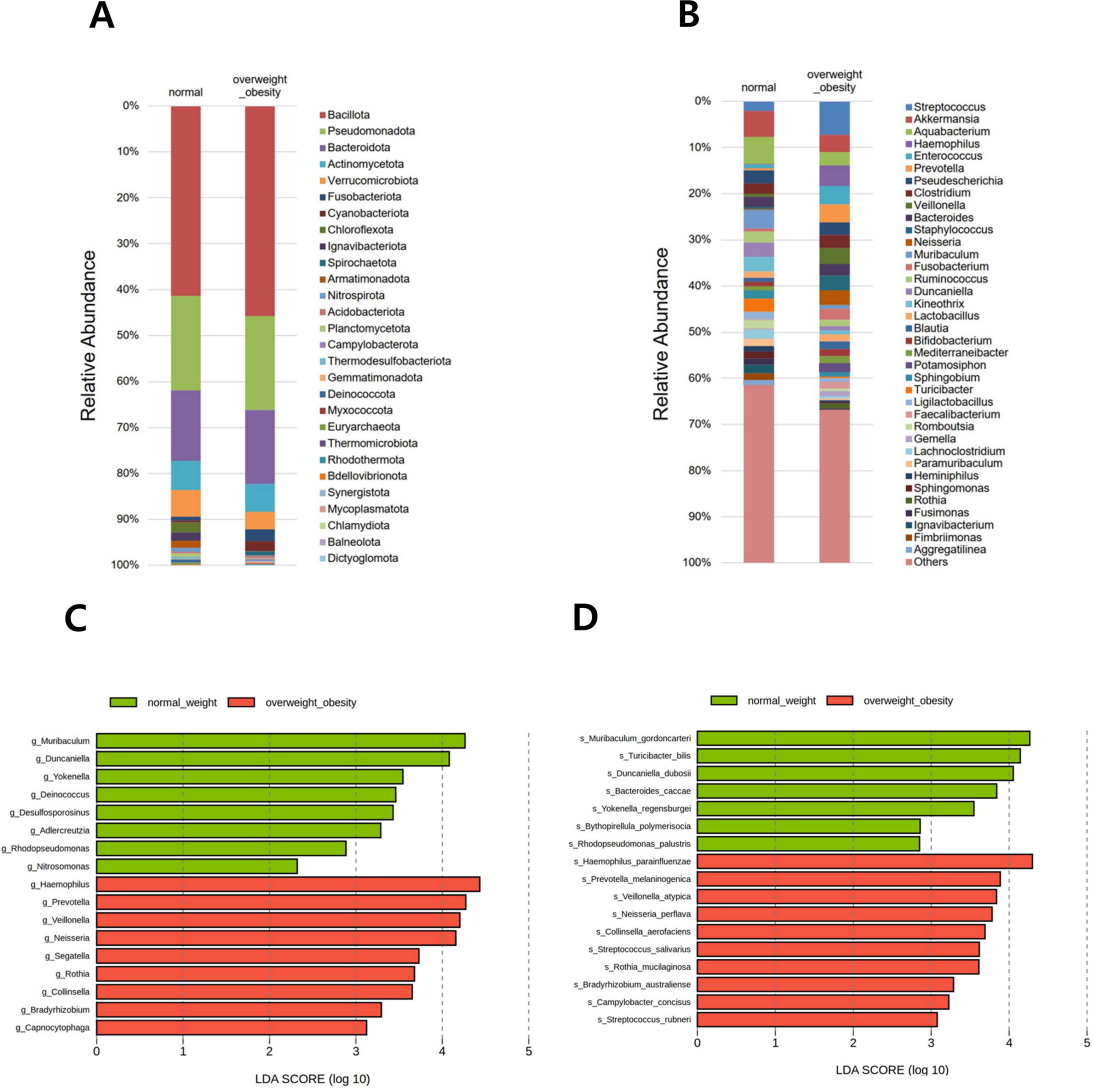
Relative abundance of the microbial community and differentially abundant taxa. Stacked bar plots show the taxonomic composition at the **(A)** phylum and **(B)** genus levels. All detected phyla are included, whereas genera are presented if the relative abundance in any group exceeded 1.0%. Differentially abundant taxa between overweight/obese and normal-weight groups were identified using linear discriminant analysis effect size (LEfSe) at the **(C)** genus and **(D)** species levels.

