# Correction: Evaluation of the gastric microbiota based on body mass index using 16S rRNA gene sequencing

**DOI:** 10.3389/fcimb.2026.1829271

**Published:** 2026-04-08

**Authors:** Sang Hoon Lee, Eun Bae Kim, Sung Chul Park, Seung-Joo Nam, Hyunseok Cho, Han Jo Jeon, Sang Pyo Lee

**Affiliations:** 1 Department of Internal Medicine, Kangwon National University College of Medicine, Chuncheon, Republic of Korea; 2 Department of Applied Animal Science, Kangwon National University College of Animal Life Sciences, Chuncheon, Republic of Korea; 3 Institute of Animal Life Science, Kangwon National University, Chuncheon, Republic of Korea; 4 Department of Pediatrics, Kangwon National University College of Medicine, Chuncheon, Republic of Korea; 5 Department of Internal Medicine, Korea University College of Medicine, Seoul, Republic of Korea; 6 Department of Internal Medicine, Hanyang University College of Medicine, Seoul, Republic of Korea

**Keywords:** body mass index, gastric microbiota, obesity, 16S rRNA sequencing, metabolic dysregulation

In the published article, an error was identified in the **Results** section regarding the description of vitamin synthesis pathway activity according to BMI group. Specifically, there was a mismatch between **Figure 4** and the corresponding **Results** description, in which vitamin synthesis pathways were incorrectly described as being more active in the normal weight group. However, as shown in **Figure 4**, the predicted functional abundances of vitamin synthesis pathways, including thiamin salvage II and tetrahydrofolate biosynthesis pathways, were higher in the overweight/obese group than in the normal weight group. This discrepancy has been corrected to ensure consistency between the figure and the **Results** section and to prevent potential misinterpretation of BMI-associated metabolic pathway activity.

A correction has been made to the **Results** section, 3.6. “*Functional profiling of gastric microbiota”*:

Instead of:

“Vitamin synthesis pathways, including superpathway of tetrahydrofolate biosynthesis, superpathway of tetrahydrofolate biosynthesis and salvage, thiamin salvage II, 6-hydroxymethyl-dihydropterin diphosphate biosynthesis I, and 6-hydroxymethyl-dihydropterin diphosphate biosynthesis III (*Chlamydia*) showed high activity in the normal weight group.”

It should be:

“Vitamin synthesis pathways, including superpathway of tetrahydrofolate biosynthesis, superpathway of tetrahydrofolate biosynthesis and salvage, thiamin salvage II, 6-hydroxymethyl-dihydropterin diphosphate biosynthesis I, and 6-hydroxymethyl-dihydropterin diphosphate biosynthesis III (*Chlamydia*) showed high activity in the overweight/obese group.”

This correction does not affect the scientific conclusions of the article in any way.

The original version of this article has been updated.

