# Supplementary figures and images for: Evaluation of the gastric microbiota based on body mass index using 16S rRNA gene sequencing
Source: Front Cell Infect Microbiol. 2025 Sep 9;15:1651316. doi: 10.3389/fcimb.2025.1651316 (PMC12454328; doi:10.3389/fcimb.2025.1651316)

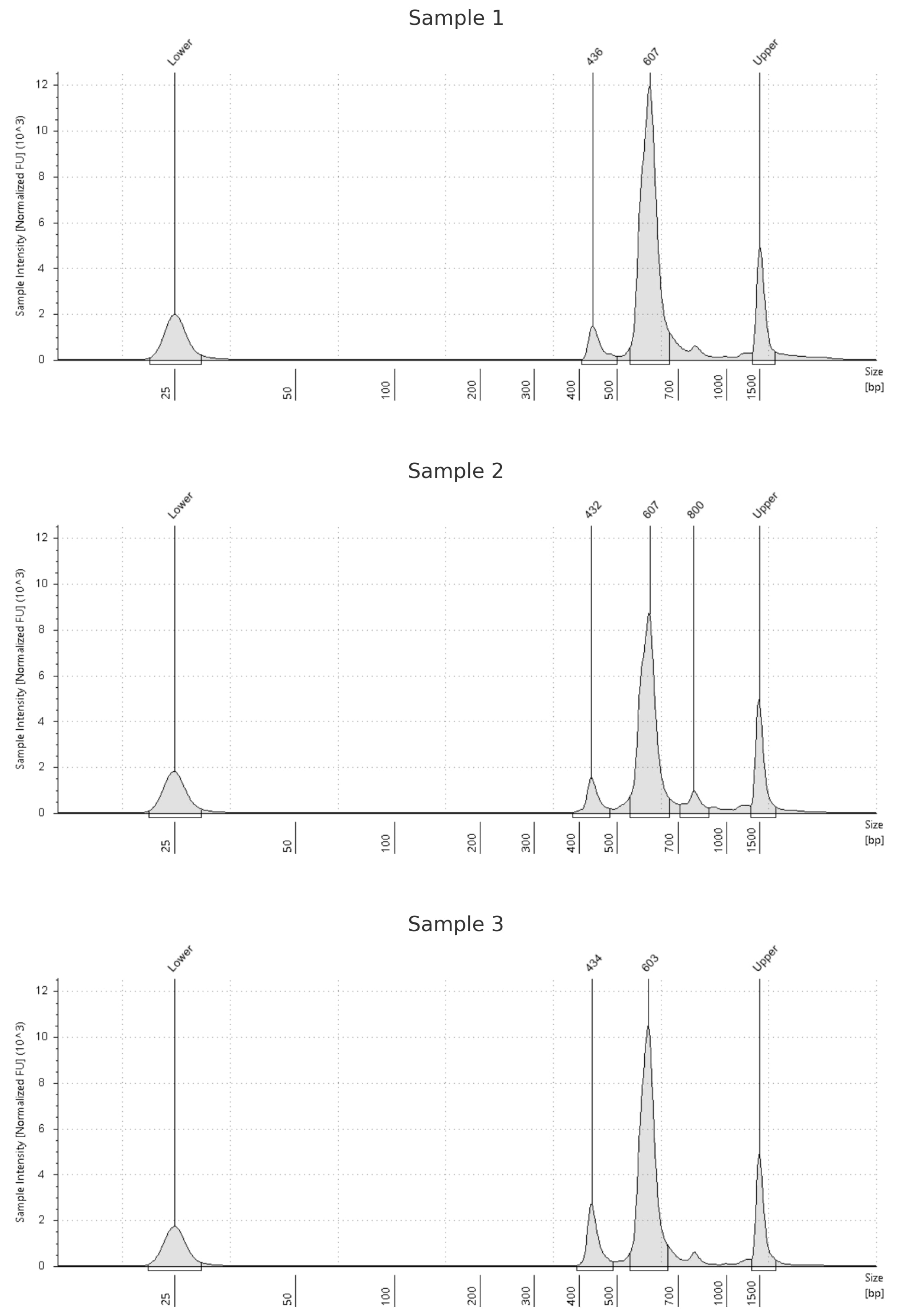

Supplement: Supplementary Figure 1 — Representative library quality control reports showing fragment size distribution of V3–V4 amplicons, confirming successful amplification and appropriate library preparation for sequencing. [file Image1.jpeg]

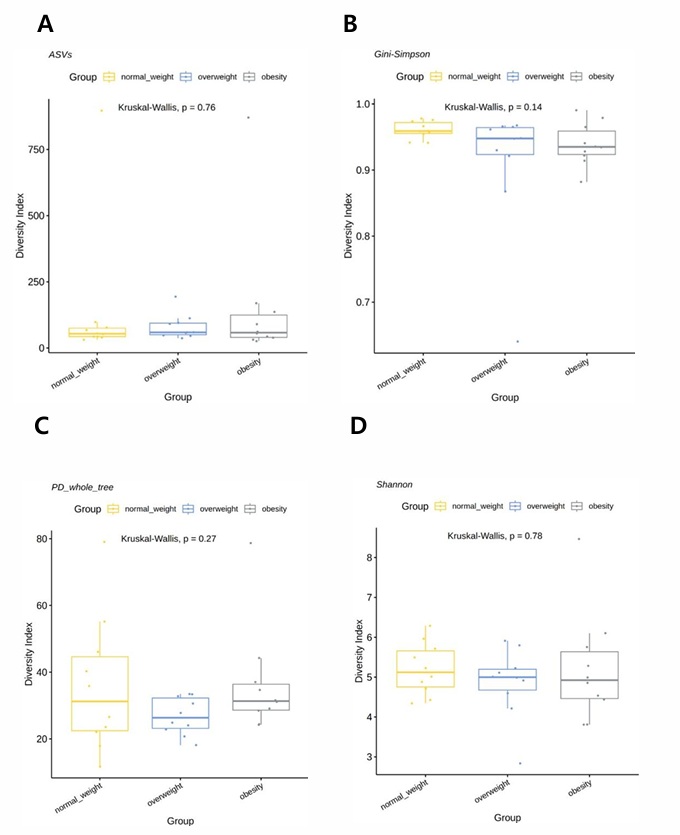

Supplement: Supplementary Figure 2 — Comparison of alpha diversity metrics of gastric microbiota among three BMI-defined groups (normal, overweight, obese). (A) Observed amplicon sequence variants (ASVs). (B) Gini–Simpson index. (C) Phylogenetic diversity (PD) whole tree. (D) Shannon index. P-values were determined using the Wilcoxon rank-sum test. [file Image2.jpeg]

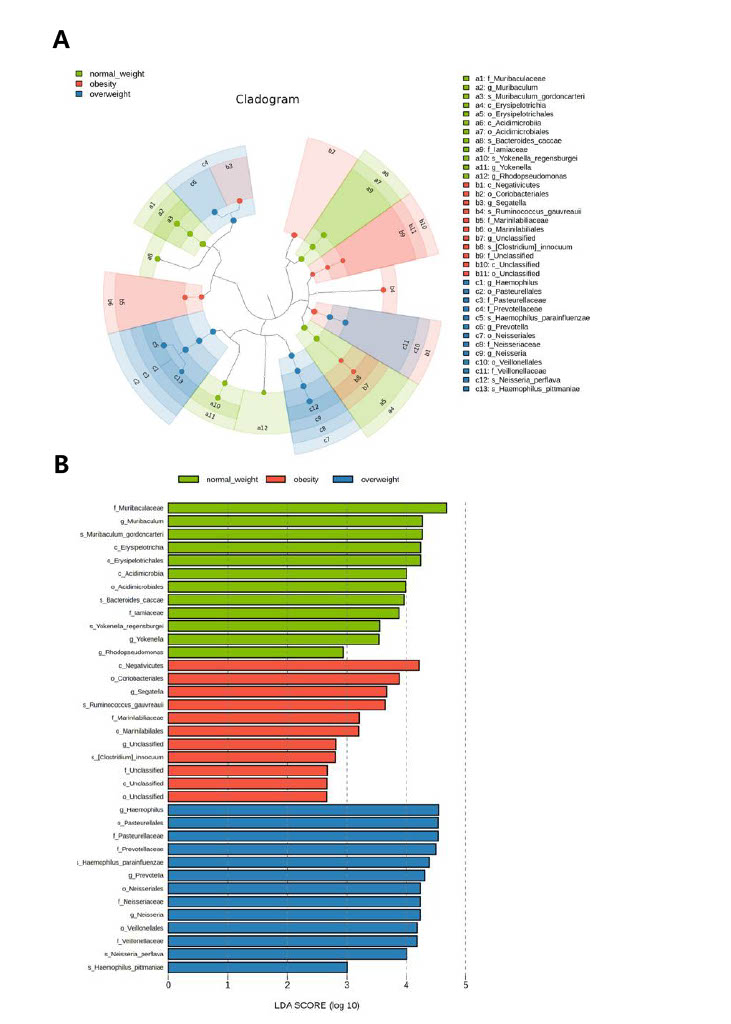

Supplement: Supplementary Figure 3 — Comparative analysis of gastric microbiota composition among normal weight, overweight, and obese groups. (A) Cladogram generated from linear discriminant analysis effect size (LEfSe) showing differentially abundant taxa across the three BMI groups (normal weight, overweight, and obese). (B) Histogram of the linear discriminant analysis (LDA) scores computed for features with differential abundance among the three BMI groups. [file Image3.jpeg]

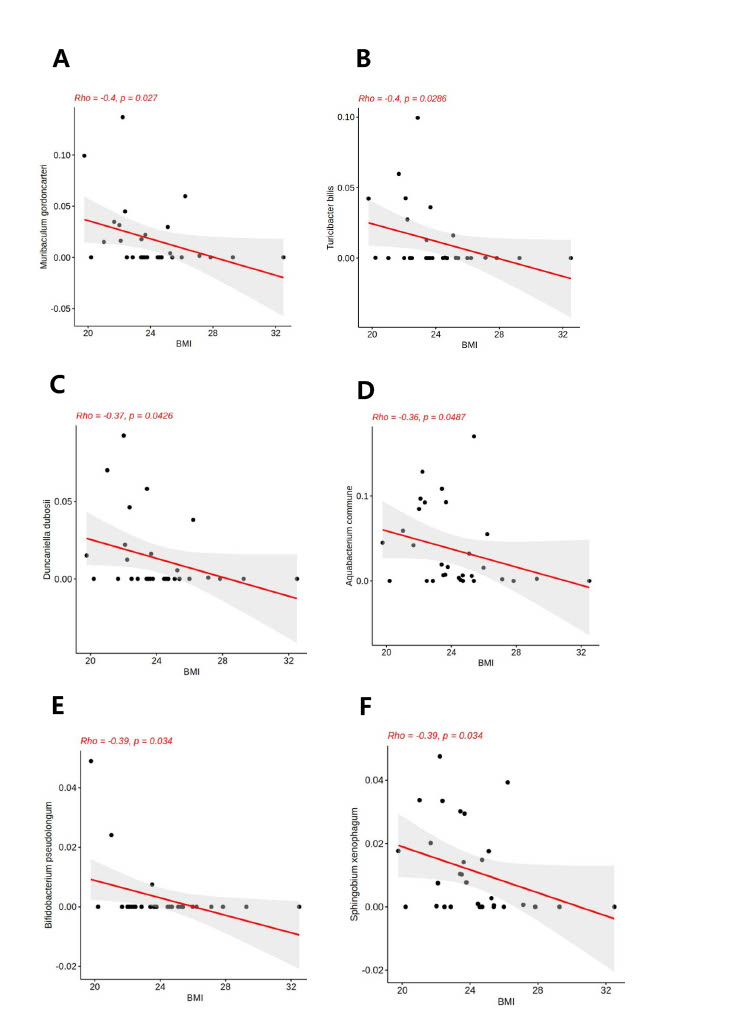

Supplement: Supplementary Figure 4 — Correlation analysis between specific bacterial species and body mass index. (A)Muribaculum gordoncarteri, (B)Turicibacter bilis, (C)Duncaniella dubosii, (D)Aquabacterium commune, (E)Bifidobacterium pseudolongum, and (F)Sphingobium xenophagum. [file Image4.jpeg]

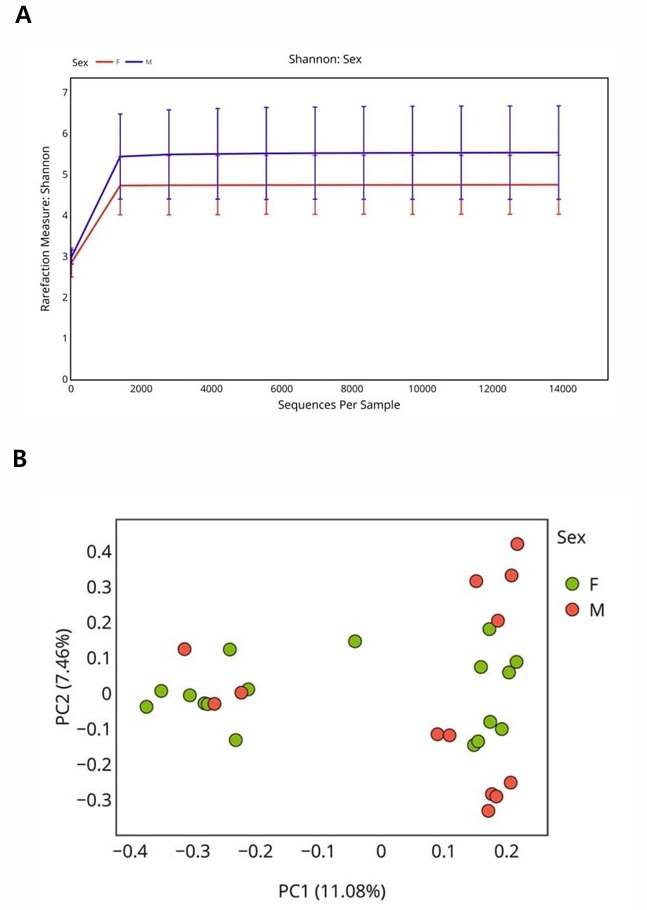

Supplement: Supplementary Figure 5 — Sex-based analysis of alpha and beta diversity in gastric microbiota. (A) Rarefaction curves of the Shannon index according to sex. (B) Principal coordinates analysis (PCoA) based on Bray–Curtis distance colored by sex. [file Image5.jpeg]
